# A highly successful case report: a patient with intestinal hepatoid adenocarcinoma and liver metastases achieves excellent efficacy following immune-targeted therapy

**DOI:** 10.3389/fonc.2026.1912875

**Published:** 2026-10-30

**Authors:** Siyu Yu, Zhenjie Dai, Dan Jiang, Hong Zhu

**Affiliations:** 1 Department of Medical Oncology, Cancer Center, West China Hospital, Sichuan University, Chengdu, Sichuan, China; 2 Department of Pathology, West China Hospital, Sichuan University, Chengdu, Sichuan, China

**Keywords:** alpha-fetoprotein, colon cancer, hepatoid colonic adenocarcinoma, immunotherapy, liver metastasis, targeted therapy

## Abstract

Hepatoid adenocarcinoma (HAC) is a rare extrahepatic adenocarcinoma producing alpha-fetoprotein (AFP), with clinicopathological features resembling hepatocellular carcinoma, including aggressive behavior, poor prognosis, vascular invasion, and distant metastasis. It occurs mostly in the stomach and rarely in the colon. Due to its rarity, large randomized trials are lacking; thus, case reports provide valuable treatment information. Here, we report a 62-year-old woman initially diagnosed with massive hepatocellular carcinoma by computed tomography (CT) who underwent radical hepatectomy. Postoperative follow-up CT revealed lesions in the sigmoid colon and residual liver. Colonoscopic biopsy showed poorly differentiated carcinoma (adenocarcinoma with enteroblastic differentiation/hepatoid adenocarcinoma). She then received CAPOX (oxaliplatin plus capecitabine) combined with sintilimab and bevacizumab. After one year, follow-up imaging showed complete disappearance of the liver tumor and reduction of the sigmoid colon lesion. The final efficacy assessment was complete remission (CR) of the liver lesion and stable disease (SD) of the sigmoid colon lesion. This case report suggests that combined immune-targeted therapy and chemotherapy achieved excellent efficacy in a patient with intestinal hepatoid adenocarcinoma and liver metastases, and this combination regimen may provide a new treatment strategy for intestinal hepatoid adenocarcinoma (IHA).

## 1 Introduction

In 1970, Bourreilile et al. (1) first described a case of liver metastasis from gastric adenocarcinoma with increased alpha-fetoprotein (AFP) in the serum. Fifteen years later Ishikura et al. (2), reporting an analog case, proposed the concept of “hepatoid carcinoma.” Since then, cases of adenocarcinoma with features mimicking hepatocellular carcinoma have been described. HAC is a rare histopathological subtype of adenocarcinoma, with an incidence ranging from 0.38% to 0.73%. HAC most commonly occurs in the digestive system, particularly in the stomach (83.9%), and less frequently in the gallbladder (3.7%), uterus (3.2%), lung (2.3%), and bladder (1.8%), among other sites (3). However, cases of colorectal HAC are extremely rare (<0.5%), and the aggressive biological behavior of this tumor leads to a very poor prognosis. It often presents at an advanced stage and is usually associated with liver metastases. AFP overproduction occurs in most but not all cases, and elevated AFP levels may help guide the diagnosis. Currently, most of the scientific literature on HAC consists of case reports or limited patient series. According to a study by Hu et al. (4) as of 2018, only 17 cases of colorectal HAC had been reported in the English literature through a PubMed search. Including their reported case, there were 12 patients with liver metastases and 11 patients with lymph node metastases. Despite aggressive treatment with radical surgery followed by adjuvant chemotherapy, 12 patients died within 12 months of diagnosis, with a median overall survival of only eight months. Thus, the treatment of this rare tumor remains challenging, and there is currently a lack of standardized treatment regimens for colorectal HAC. Herein, we present a case report of a patient with sigmoid colon HAC with liver metastases. After one year of treatment with the CAPOX regimen combined with bevacizumab and sintilimab, the patient achieved remarkable tumor regression: the liver metastases completely disappeared, the primary sigmoid colon lesion shrank, and the surrounding lymph nodes shrank or even disappeared. Only mild side effects occurred during treatment.

## 2 Case presentation

A 62-year-old female patient presented with right-sided abdominal pain and underwent CT and magnetic resonance imaging (MRI) at another hospital, which revealed an abnormal space-occupying lesion in the right lobe of the liver. She was subsequently referred to our hospital for further evaluation. Upper abdominal CT at our institution showed a large, mixed-signal mass lesion in the right hepatic lobe, measuring approximately 10.9 × 8.1 cm (**Figure 1A**). The mass in the right hepatic lobe along with a nodule in the adjacent subsegment of the right posterior inferior lobe were highly suggestive of hepatocellular carcinoma (HCC). The serum AFP level was 181,968 ng/mL. Based on the imaging findings and tumor marker results, the initial diagnosis was HCC. On February 21, 2025, the patient underwent open right-sided complex hepatectomy (H1’5678), cholecystectomy, partial diaphragmatic resection, portal vein repair, and vena cava repair. Postoperative pathology indicated poorly differentiated carcinoma. The tumor cells were arranged in a trabecular pattern with round to ovoid nuclei, some with intranuclear pseudoinclusions and prominent nu cleoli, and abundant eosinophilic cytoplasm resembling the morphology of a hepatocellular carcinoma (**Figures 2a, b**).

**Figure 1:**
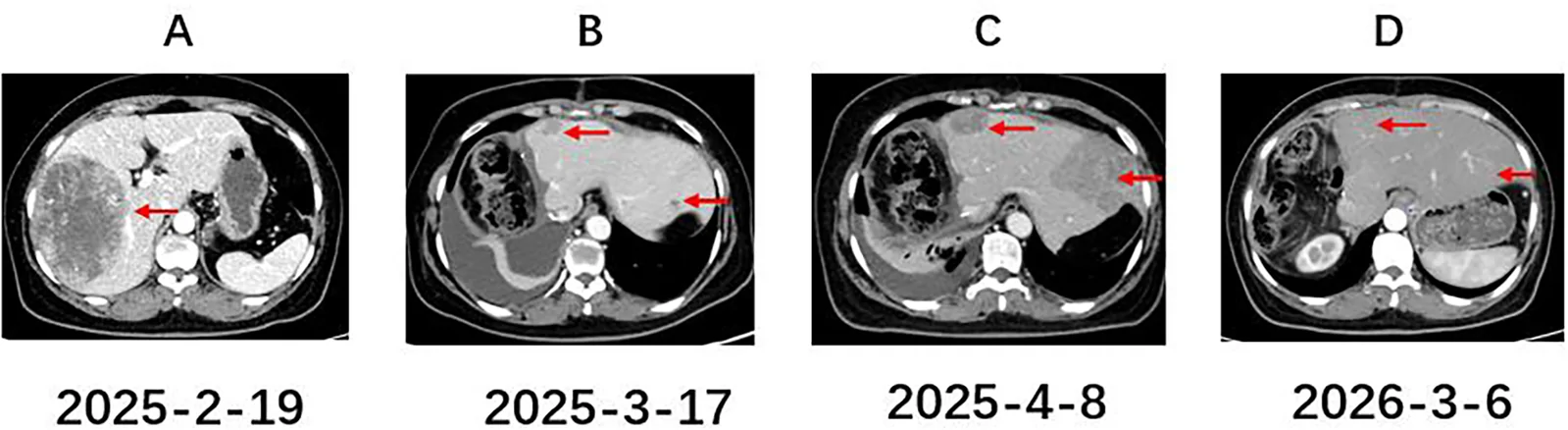
Representative CT images of liver metastases at different stages before and after treatment. Red arrows indicate hepatic metastases. **(A)** CT imaging on February 19, 2025, showed a massive liver metastasis. **(B)** CT scan obtained 3 weeks after surgery showed new lesions in the residual liver, suggestive of tumor recurrence. **(C)** Imaging showed continued enlargement of the lesions in the residual liver, consistent with progressive disease (PD). **(D)** Imaging showed complete disappearance of the liver metastases.

**Figure 2:**
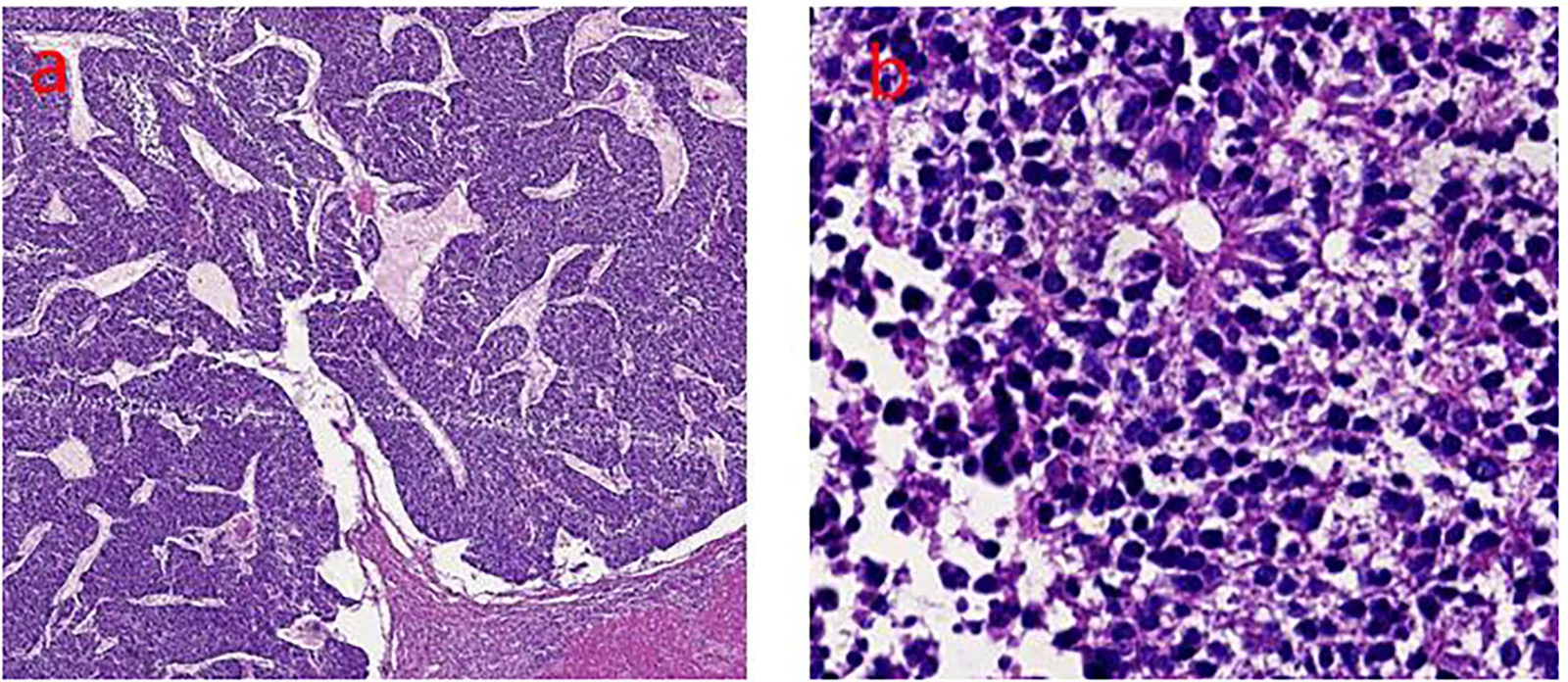
Histopathology of the resected liver showing a poorly differentiated carcinoma with hepatoid features. **(a)** H&E stain, cale bar = 250μm. **(b)** H&E stain, cale bar = 10μm.

However, a follow-up CT 3 weeks after surgery revealed new lesions in the residual liver that rapidly enlarged (**Figures 1B, C**). Subsequent colonoscopy confirmed a sigmoid colon tumor, and pathological biopsy indicated adenocarcinoma with enteroblastic differentiation/hepatoid adenocarcinoma. Microscopic evaluation revealed that the majority of tumor cells exhibited a large polygonal shape with abundant eosinophilic cytoplasm, large and deeply stained nuclei, evident heterogeneity, and the presence of eosinophilic vesicles in the cytoplasm (**Figures 3A, B**). Immunohistochemistry showed that the tumor cells were positive for SATB2 (**Figure 4a**), CDX2 (+) (**Figure 4b**), Villin (+) (**Figure 4c**), SALL4 (+) (**Figure 4d**), GPC3 (+) (**Figure 4e**), and AFP (**Figure 4f**). CDX2 positivity suggested a primary intestinal origin. Based on the morphological features, immunohistochemical markers, and molecular testing, the diagnosis was colorectal hepatoid adenocarcinoma with liver metastases.

**Figure 3:**
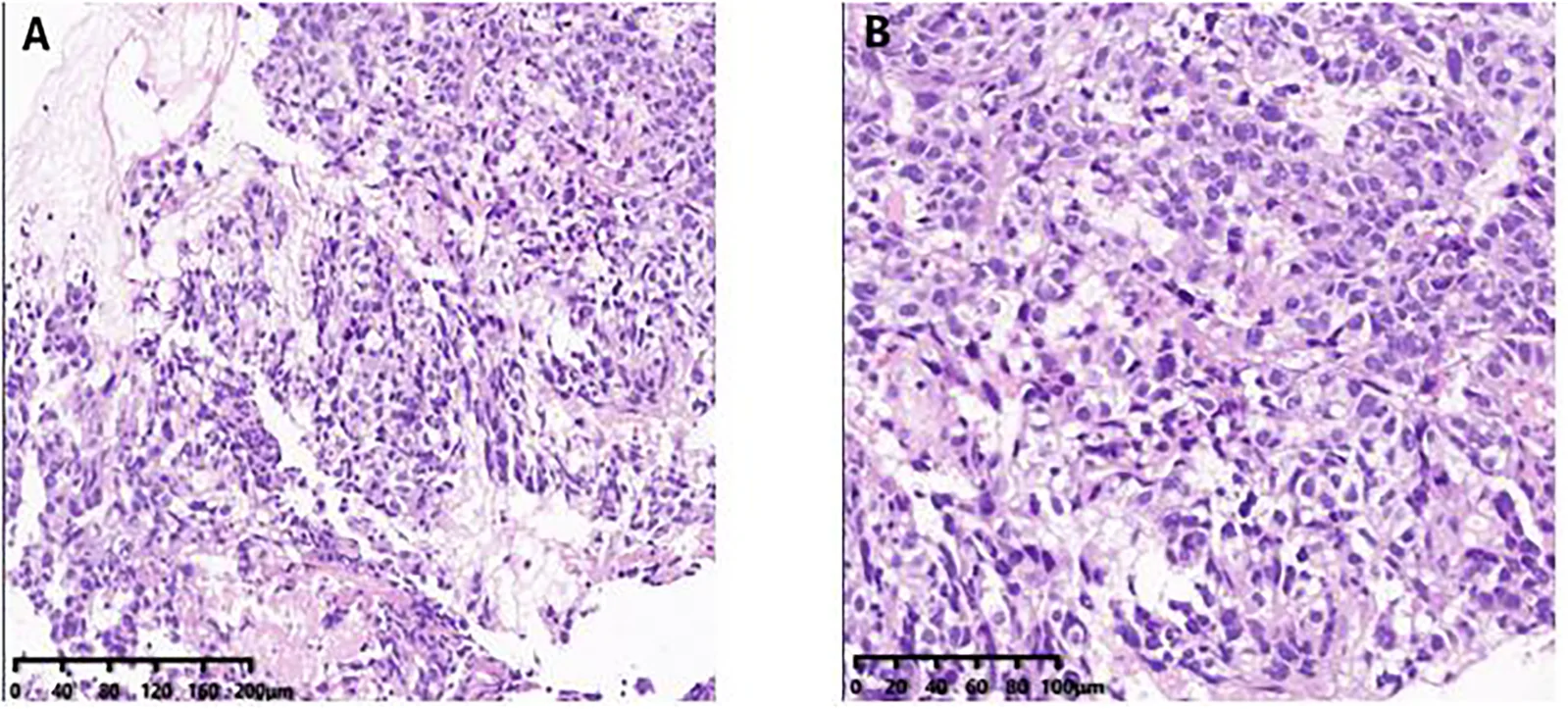
Hematoxylin and eosin staining: the majority of tumor cells exhibited a large polygonal shape with abundant eosinophilic cytoplasm, large and deeply stained nuclei, evident heterogeneity, and presence of eosinophilic vesicles in the cytoplasm. **(A)** H&E stain, scale bar = 40μm. **(B)** H&E scale bar = 20μm.

**Figure 4:**
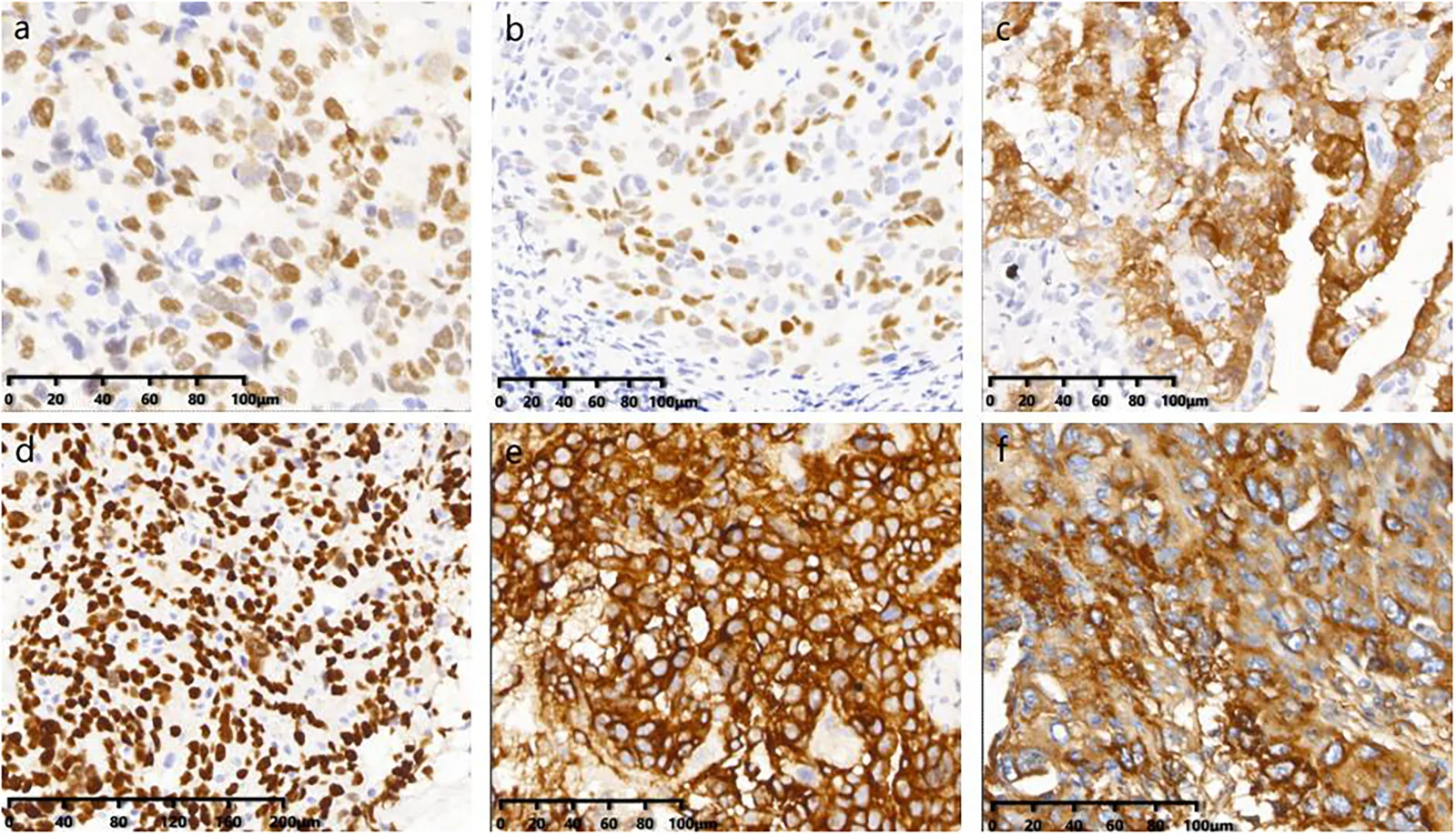
Immunohistochemistry showing that the tumor cells are positive for SATB2 **(a)**, CDX2 (+) **(b)**, Villin (+) **(c)**, SALL4 (+) **(d)**, GPC3 (+) **(e)**, and AFP **(f)** [**(a–f)**, scale bar = 20μm].

Given the extreme rarity of IHA, no specific treatment regimen is currently available in the literature. In general, the treatment strategy for IHA is similar to that for conventional adenocarcinoma (CA). CAPOX is a standard first-line regimen for colorectal cancer, and capecitabine offers the convenience of oral administration and a manageable safety profile. Therefore, the patient received treatment with the CAPOX regimen combined with sintilimab and bevacizumab, with sintilimab and bevacizumab added starting from the second cycle of chemotherapy. Before treatment, the patient’s serum AFP level was extremely high (181,968 ng/mL). Three months after initiating treatment for the colorectal lesion and liver metastases (i.e., after three cycles of chemotherapy combined with immunotherapy and targeted therapy), the serum AFP level decreased dramatically to 137 ng/mL (**Figure 5**). After completion of four cycles of chemotherapy, the patient received maintenance therapy with sintilimab and bevacizumab. Follow-up whole-abdominal CT on March 6, 2026, showed complete disappearance of the residual liver mass, marked reduction in the size of the sigmoid colon lesion (**Figures 1D**, **6**). Telephone follow-up revealed that the patient’s serum AFP level, measured at another hospital on May 8, 2026, had stabilized at 0.6 ng/mL. The latest follow-up was conducted by telephone on August 24, 2026, at which time the serum AFP level was 0.8 ng/mL. Although the patient had very advanced-stage disease and experienced rapid recurrence and metastasis, she achieved remarkable efficacy with this regimen, with only mild side effects and no severe post-chemotherapy bone marrow suppression or other adverse reactions. At present, the patient’s progression-free survival is approximately 16 months, and the current overall survival is approximately 18 months. The patient has a good health, and we, the investigators, will continue longer-term follow-up to confirm the durability of the remission. The timeline of the patient's treatment and clinical course is summarized in **Figure 7**.

**Figure 5:**
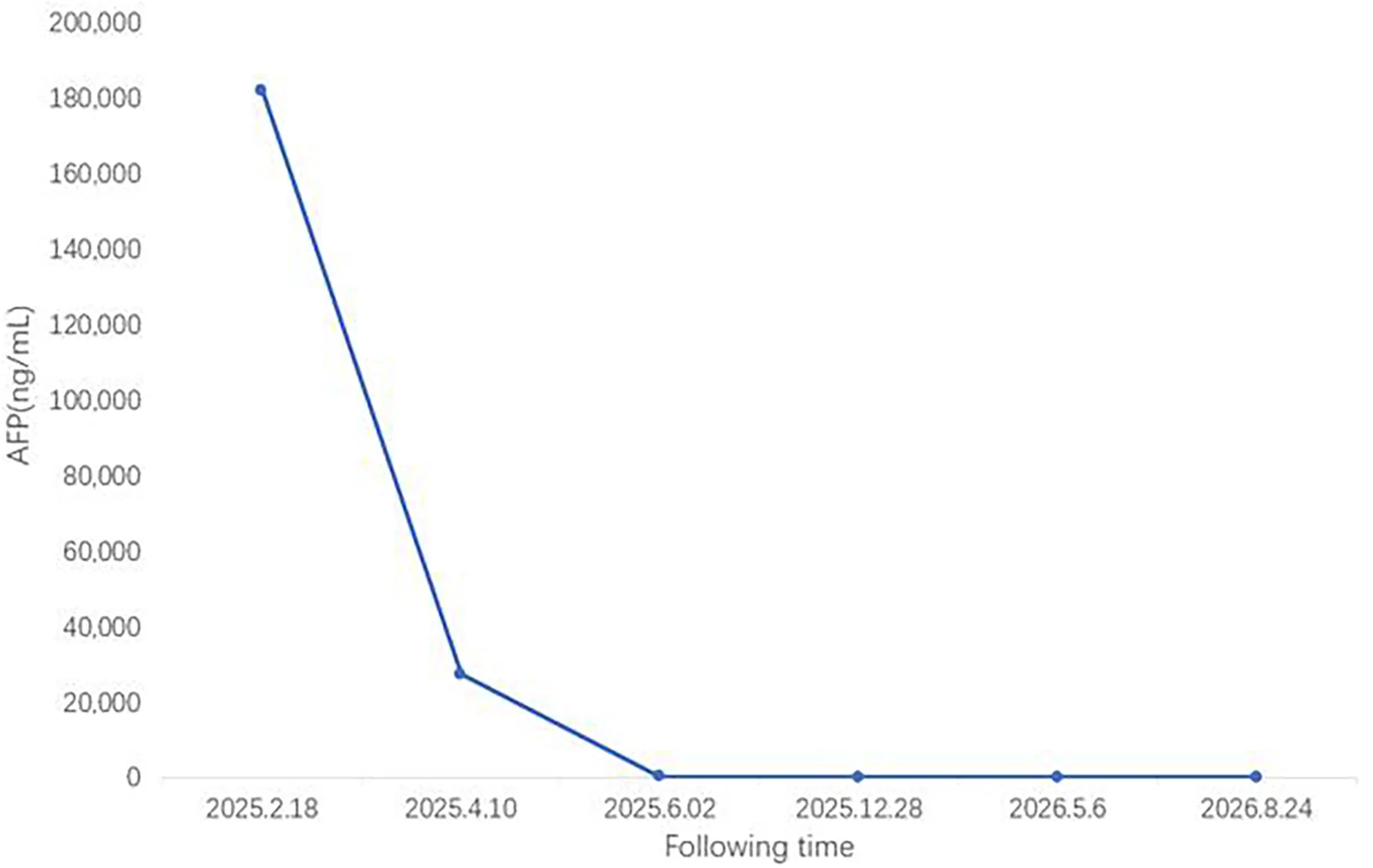
Level of serum alpha-fetoprotein (AFP). After the patient underwent open right-sided complex hepatectomy, the serum alpha-fetoprotein (AFP) level decreased from 181,968 ng/mL (February 18, 2025) to 27,696 ng/mL (April 10, 2025). Subsequently, after three cycles of the CAPOX regimen combined with immunotherapy and targeted therapy, it decreased to 137 ng/mL (June 2, 2025). Currently, the patient’s AFP level has stabilized below 1 ng/mL.

**Figure 6:**
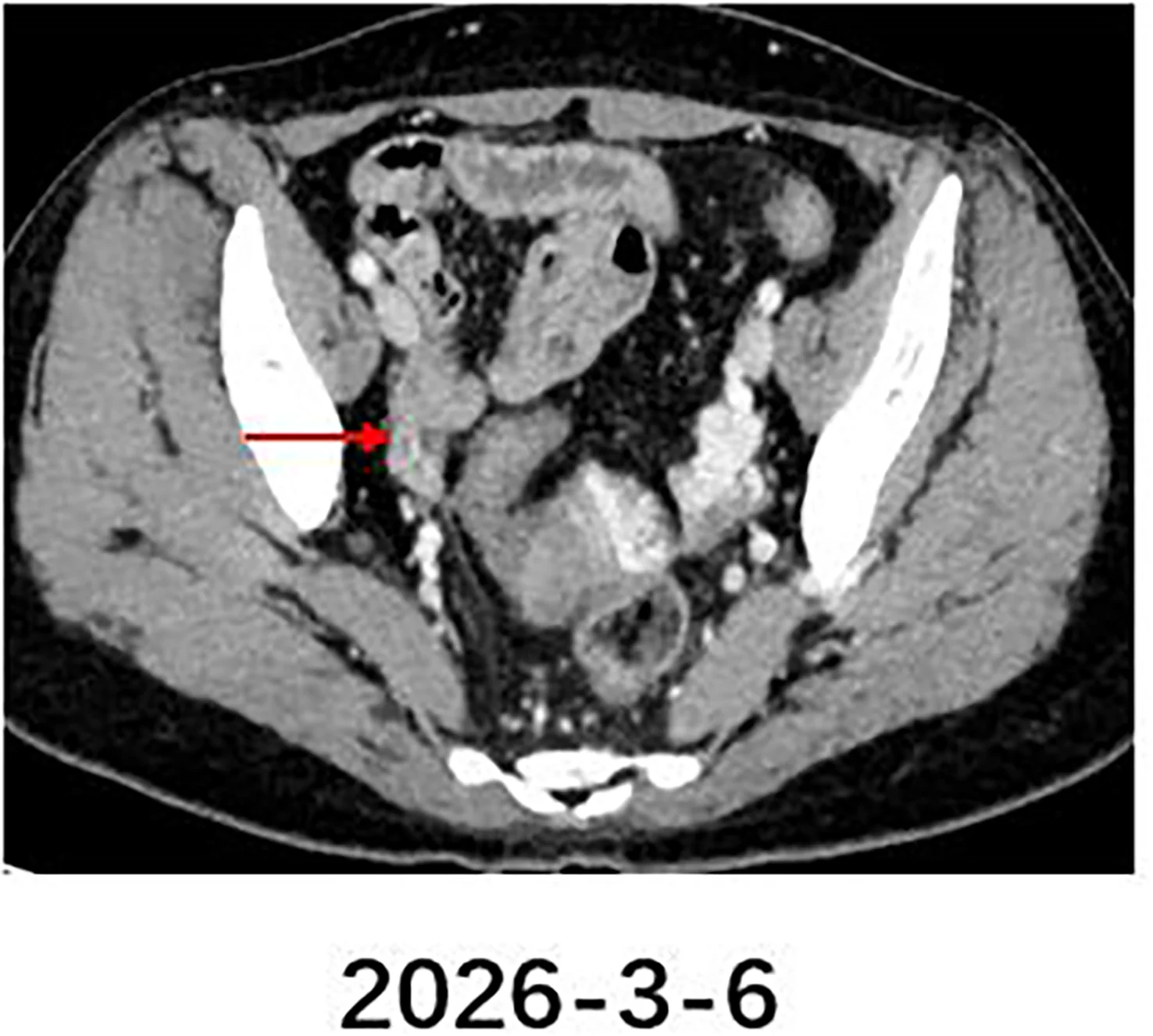
Representative post-treatment CT image of the sigmoid colon tumor. Red arrows indicate the tumor location.

**Figure 7:**
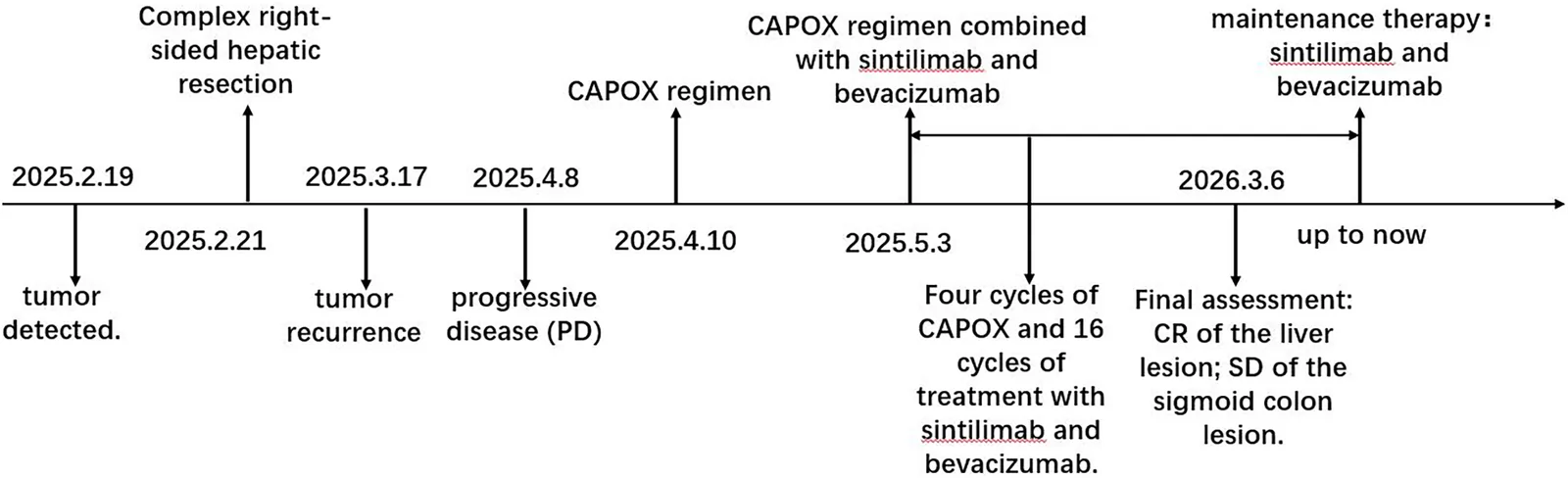
Timeline of patient treatment.

## 3 Discussion

Intestinal hepatoid adenocarcinoma (IHA) is a rare and distinct malignant tumor of the intestine. It is often characterized by elevated serum AFP, hepatoid morphology, poor prognosis, and expression of hepatocyte markers such as AFP, HepPar-1, and GPC3. Colorectal-specific markers, such as CDX2, CK20, and SATB2, help confirm the primary tumor origin and distinguish IHA from metastatic hepatocellular carcinoma or other malignancies. Early diagnosis is crucial for improving the prognosis of patients with IHA, but preoperative definitive diagnosis is extremely challenging. First, clinically, IHA is often associated with elevated serum AFP levels and exhibits extensive vascular invasion, frequent liver metastases, and usually an advanced pTNM stage. All of these factors contribute to its extremely poor prognosis (5). Zeng et al. (6) reviewed 42 patients with IHA and found that IHA most commonly occurred in the colon, followed by the rectum. Most patients presented with bloody stools, especially in cases involving the colorectum. In addition, the majority of IHA cases were associated with elevated serum AFP levels, with some even showing extremely high levels, which facilitates objective diagnosis (7, 8). Its overproduction is neither a necessary condition for diagnosis nor related to prognosis. Secondly, the imaging findings of IHA are non-specific: it typically presents as a large mass with a density similar to that of normal liver on baseline imaging, and shows moderate enhancement after intravenous administration of iodinated contrast medium. These findings do not differ significantly from other malignant tumors (9, 10). Furthermore, even preoperative biopsy rarely establishes a definitive diagnosis of IHA. In terms of treatment, due to the extreme rarity of IHA, there are currently no specific treatment data available in the literature. Generally, the treatment strategy for IHA is similar to that for conventional adenocarcinoma (CA), and radical surgery combined with simultaneous resection of liver metastases, followed by systemic chemotherapy, is considered the optimal choice (11).

However, compared with CA, IHA has a higher rate of lymph node metastasis and more frequent distant metastasis, making radical resection more challenging. In terms of prognosis, IHA has an extremely poor prognosis, far worse than that of CA. Approximately half of the patients die within one year after diagnosis, with 1-, 2-, and 3-year disease-specific survival (DSS) rates are 30.6%, 22.9%, and 7.6%, respectively, whereas those for CA are as high as 84.7%, 76.6%, and 70.4%, respectively (6). The study found that only pTNM stage was an independent risk factor for both progression-free survival (PFS) and DSS, while chemotherapy showed no significant correlation with either. This indicates that IHA is not sensitive to conventional chemotherapy drugs used for colorectal cancer, and its biological behavior is more similar to that of primary liver tumors. In terms of primary tumor origin tracing, Hu et al. (4) while investigating the origin of HAC, found that CDX2 was positive in their case. CDX2 is a relatively specific marker of intestinal origin for adenocarcinoma and can be used as part of a panel to determine the origin of metastatic adenocarcinoma (12). In addition, SATB2 and CK20 were also positive. SATB2 is a biomarker for colorectal cancer (CRC), with approximately 85% of CRC patients testing positive, while its expression is rare in other cancer types. The combination of SATB2 and CK20 can identify over 95% of colorectal carcinomas (13). In the present case, both CK20 and SATB2 were positive, further supporting this view.

Here, we present a patient with intestinal HAC and liver metastases. Despite radical resection of the liver metastasis, the liver tumor recurred rapidly. In this case, the recurrence may be related to several factors: first, at the time of hepatectomy, the primary sigmoid colon tumor had not yet been identified and may therefore have continued to seed the liver; second, HAC is highly aggressive and prone to vascular invasion and early distant metastasis; in addition, surgery itself may transiently promote a pro-inflammatory and immunosuppressive environment that facilitates the outgrowth of residual micrometastases. After four cycles of the CAPOX regimen, the patient received maintenance therapy with bevacizumab and sintilimab up to the present. The latest imaging studies demonstrated complete disappearance of the liver metastases, as well as shrinkage or even disappearance of the primary lesion and surrounding lymph nodes. After three cycles of treatment, the serum AFP level decreased dramatically from 181,968 ng/mL in February 2025 to 137 ng/mL in June 2025. Due to the extreme rarity of this disease, the current treatment strategy for colorectal HAC is similar to that for conventional colorectal adenocarcinoma, mainly including surgical resection of the primary tumor and selected metastatic lesions, systemic chemotherapy, and palliative care. Among these, radical surgery is considered the preferred treatment option to prolong patient survival (14). For patients who are not candidates for radical resection, systemic therapy remains the cornerstone of treatment. Regarding the choice of chemotherapy regimen, most of the successfully treated cases of HAC reported in the literature have adopted platinum-based chemotherapy (15). For example, some researchers used hepatic arterial infusion chemotherapy (HAIC) with doxorubicin combined with oxaliplatin, which successfully and significantly reduced the patient’s serum AFP level. Subsequently, the use of the folinic acid, fluorouracil, and oxaliplatin (FOLFOX) regimen combined with epirubicin and capecitabine as maintenance therapy also showed good efficacy in preventing recurrence (16).

In addition, some studies have combined bevacizumab with FOLFOX or FOLFIRI (leucovorin, 5-fluorouracil, irinotecan) regimen. For example, Yuanyuan et al. reported a 36-year-old male patient with disease recurrence who, after receiving FOLFOX plus bevacizumab, experienced a significant decrease in serum AFP levels and achieved stable disease at six months (17). In that case, the investigators used platinum-based chemotherapy combined with targeted therapy, and the patient achieved stable disease with a significant decline in serum AFP. This suggests that, in addition to platinum-based chemotherapy, targeted therapy may also confer clinical benefit in patients with HAC. However, because this was only a single case report and the patient received a combination regimen, the extent to which targeted therapy contributed remains uncertain, and further clinical studies are warranted. It is noteworthy that, compared with other cancers, HAC exhibits more abundant angiogenesis, and the majority of cancer cells express vascular endothelial growth factor (VEGF), which is a key factor promoting angiogenesis *in vitro* and *in vivo*. Therefore, inhibiting tumor angiogenesis and downregulating VEGF expression with effective drugs may be an important strategy for targeted therapy in HAC. Bevacizumab, as the first anti-angiogenic drug approved for cancer treatment, primarily targets VEGF. By inhibiting VEGF activity, it blocks tumor neovascularization, thereby suppressing tumor cell proliferation and exerting an antitumor effect (18). In the present case, the patient received a platinum-based chemotherapy regimen combined with bevacizumab and sintilimab, and after a period of treatment, the serum AFP level also decreased significantly. This suggests that platinum-based chemotherapy combined with anti-VEGF targeted therapy may exert a synergistic antitumor effect in HAC and may contribute to the complete remission (CR) observed in this case.

Since the advent of immune checkpoint inhibitors, immunotherapy with checkpoint inhibitors has greatly changed the treatment landscape for many types of malignant tumors and brought significant survival benefits. However, data on immune checkpoint blockade therapy in HAC are extremely limited, and treatment strategies are largely based on case reports or small-sample studies, making it difficult to draw definitive conclusions regarding the optimal immunotherapy regimen. A recent comprehensive analysis of cases of hepatoid adenocarcinoma of the lung (HAL) showed that HAL generally has limited sensitivity to conventional chemotherapy, whereas combination therapy with agents targeting specific molecules or integrated immunotherapy appears to significantly improve patient prognosis (19).This suggests that combining immunotherapy with other treatment modalities may also confer survival benefits in HAC arising at other sites. In a case of hepatoid adenocarcinoma of the stomach (HAS) reported by Li et al. (20), a 64-year-old male patient received neoadjuvant chemo-immunotherapy consisting of oxaliplatin, S-1, and sintilimab. Over a 2-month period, the patient’s serum AFP level decreased significantly from 52,951.56 ng/mL to 241.04 ng/mL, and CT scans revealed substantial tumor regression; subsequent radical surgery confirmed significant tumor shrinkage with no evidence of lymph node metastasis. This case shares a treatment strategy consistent with ours, as both employed platinum/fluoropyrimidine-based chemotherapy combined with a PD-1 inhibitor. In the present case, however, anti-VEGF targeted therapy (bevacizumab) was added to the regimen, and complete remission (CR) of liver metastases was achieved in the context of rapid recurrence after radical surgery, which is even rarer among previously reported cases of gastric HAS.

HAS typically exhibits an “immunologically cold” phenotype. An immunomicroenvironment analysis of 65 HAS cases confirmed sparse immune infiltration, with immune cells mainly confined to the periphery of gastric adenocarcinoma glands, while almost no lymphocyte infiltration was observed in areas of hepatocellular carcinoma-like differentiation; therefore, the predictive value of conventional biomarkers is limited (21). However, the remarkable efficacy in this case suggests that chemotherapy combined with anti-VEGF therapy may overcome the inherent resistance of HAC to immunotherapy by remodeling the tumor microenvironment (TME). Oxaliplatin can induce immunogenic cell death and activate the cGAS-STING pathway, increasing cytotoxic T-cell infiltration (22). Bevacizumab improves tumor vasculature and hypoxia by inhibiting VEGF, promoting immune cell infiltration. Together, they create favorable conditions for the PD-1 inhibitor (sintilimab) to relieve immunosuppression and activate T-cell killing function (18, 22, 23). A case report of gastric HAC showed that pembrolizumab combined with bevacizumab and chemotherapy as second-line treatment achieved a progression-free survival of 14 months and an overall survival of 16 months, with tolerable adverse effects, further supporting the clinical value of this combination strategy in HAC (24). In the present case, AFP decreased from 181,968 ng/mL to 137 ng/mL, highly consistent with complete remission on imaging, further supporting the value of dynamic AFP monitoring in evaluating treatment response in HAC.

In the present case, the patient received platinum-based chemotherapy combined with bevacizumab and sintilimab, and is currently still receiving maintenance therapy with sintilimab and bevacizumab. The latest CT imaging showed complete disappearance of the liver metastases. Assessment based on RECIST v1.1 showed that the treatment response reached CR. Therefore, we consider that the favorable treatment outcome in this patient may be associated with the combination of targeted therapy and immunotherapy. This provides a new treatment option for IHA and offers clinical evidence supporting individualized combination strategies.

## 4 Conclusion

IHA is a very rare subtype of intestinal cancer. At diagnosis, lymph node and distant organ metastases, especially liver metastases, are often present, posing a major therapeutic challenge and resulting in poor treatment outcomes. This case report suggests that the combination of bevacizumab and sintilimab with chemotherapy is an effective and safe regimen for IHA. In particular, combined immunotherapy and targeted therapy may serve as an effective first-line treatment option for IHA. Future studies should evaluate this regimen in larger cohorts or randomized controlled trials to provide stronger evidence for the treatment of IHA.

## Data Availability

The datasets presented in this study can be found in online repositories. The names of the repository/repositories and accession number(s) can be found in the article/supplementary material.
